# Distribution of Spinal Sensitization Evoked by Inflammatory Pain Using Local Spinal Cord Glucose Utilization Combined with ^**3**^H-Phorbol 12,13-Dibutyrate Binding in Rats

**DOI:** 10.1155/2013/340167

**Published:** 2013-12-26

**Authors:** Yasuda Seiko, Ishikawa Kozo, Matsumoto Yoshihiro, Ariyoshi Toru, Sasaki Hironori, Ida Yuika, Iwanaga Yasutake, Kim Hae-Kyu, Nakanishi Osamu, Ishikawa Toshizo

**Affiliations:** ^1^Division of Neurosciences and Department of Laboratory Sciences, Yamaguchi University Graduate School of Medicine, 1-1-1 Minami-Kogushi, Ube, Yamaguchi 755-8505, Japan; ^2^2nd Department of Anatomy, Sapporo Medical University, 17-Nishi Minami-1, Chuo-ku, Sapporo 060-8556, Japan; ^3^Department of Dental Anesthesiology, Kyushu Dental University, 3-1-6 Manazuru, Kokura, Kita-kyusyu 803-0001, Japan; ^4^Department of Anesthesiology, Pusan National University, 9 Bugok 3-dong, Geumjeong-gu, Busan 609-757, Republic of Korea

## Abstract

*Aims*. Hyperalgesia following tissue injury is induced by plasticity in neurotransmission. Few investigators have considered the ascending input which activates the superficial of spinal cord. The aim was to examine neurotransmission and nociceptive processing in the spinal cord after mustard-oil (MO) injection. Both *in vitro* and *in vivo* autoradiographs were employed for neuronal activity and transmission in discrete spinal cord regions using the ^14^C-2-deoxyglucose method and ^3^H-phorbol 12,13-dibutyrate (^3^H-PDBu) binding sites. *Methods*. To quantify the hyperalgesia evoked by MO, the flinching was counted for 60 min after MO (20%, 50 *μ*L) injection in Wistar rats. Simultaneous determination of ^14^C-2-deoxyglucose and ^3^H-PDBu binding was used for a direct observation of neuronal/metabolic changes and intracellular signaling in the spinal cord. *Results*. MO injection evoked an increase in flinching for 60 min. LSCGU significantly increased in the Rexed I-II with ^3^H-PDBu binding in the ipsilateral side of spinal cord. *Discussion*. We clearly demonstrated that the hyperalgesia is primarily relevant to increased neuronal activation with PKC activation in the Rexed I-II of the spinal cord. In addition, functional changes such as “neuronal plasticity” may result in increased neuronal excitability and a central sensitization.

## 1. Introduction

A number of reports have characterized the mechanisms underlying pathological pain, primarily by investigating pain-producing substances in peripheral sensory receptors and pain transmitters in the spinal cord, as well as the associated pain pathways. More recent attempts have gradually increased understanding through the investigation of neuroglial interactions and spinal sensory nerves and the modulation of spinal pain transmission in relation to refractory hyperalgesia. It has been suggested that following tissue damage/inflammation, repetitive pain receives contributions from neuronal plasticity in the processes of nociception.

Repetitive noxious stimulation to primary afferent C fibers produces a sustained hyperalgesia [1] leading to exaggerated pain-related behavior, for example, shaking or flinching (“hyperalgesia”). Woolf and Salter [2] proposed that neuroplastic changes in spinal synaptic transmission are the primary mechanism behind persistent spontaneous pain and hyperalgesia following such peripheral tissue and nerve injury. Ample research conducted subsequently suggests that mitogen associated protein kinase family; MAPKs (extracellular signal-regulated kinase 1/2: ERK1/2, p38-MAPK, and c-jun N-terminal kinase: JNK) [3, 4] and brain-derived neurotrophic factor (BDNF) derived from glial cell activation contribute to these neuroplastic changes [5]. A variety of intracellular signaling processes are modified during the progression of pain. Zhuang et al. [6] investigated the contributions of pERK as a marker and proposed a profile based on their findings. However, few investigators have delineated pain pathways conclusively.

Subcutaneous injection of irritating substances such as formalin or mustard oil (MO) into the rat hind paw produces sustained flinching [7]. In MO-induced model, continuous stimulation of pain receptors by neurogenic inflammation induced hyperalgesia in the acute and subacute stages of tissue inflammation. The model used was, therefore, well suited to the analysis of molecular changes over time in pain response and was fit for the objectives of this study. Therefore, this model is appreciated for clinical applicability, exhibiting symptoms similar to those associated with traumatic tissue injury in humans. Sustained afferent input as initiated by the intraplantar injection of MO yields an increased release of substance P (sP) and excitatory amino acids (EAAs). It also evokes a sustained activation of N-methyl-D-aspartate (NMDA) receptor, leading to a signaling cascade [8]. This cascade includes increased prostaglandins E_2_ (PGE_2_) and nitric oxide release, with the activation of protein kinase C (PKC) [9–11]. Support for the role of NMDA in this cascade is provided by the observation that pain behavior is attenuated by intrathecal (i.t.) NMDA antagonists. Increases in intracellular Ca^2+^ occur secondary to depolarization and the opening of calcium ionophores such as the NMDA receptor. This may activate the Ca^2+^-dependent enzyme, PKC, which is known to enhance neuronal excitability and is associated with “long-term potentiation” in the hippocampus. It has thus been suggested that activation of PKC may also cause long lasting firing of dorsal horn neurons of spinal cord. This results in the development of hyperalgesia [12]. However, direct evidence demonstrating functional changes (neuronal activity) along with synaptic transmission in discrete regions of the spinal cord during hyperalgesia is still lacking.

Thus, we aimed to examine pain-related response in relation to synaptic transmission in a discrete spinal cord region produced by peripheral paw injection of MO and its nociceptive processing in the spinal cord in rats. To elucidate mechanisms involved in pain-related behavior, we employed a series of experiments using the simultaneous determination of ^14^C-2-deoxyglucose [13] method and *in vitro* auto-radiography to investigate the binding site of ^3^H-phorbol 12,13-dibutyrate (^3^H-PDBu) [14]. Using these methods, we provide evidence for the spatial distribution of neuronal activity in the spinal cord and PKC activation/translocation involved in pain pathways.

## 2. Materials and Methods

The experimental protocol used in this study was approved by the Ethics Committee for Animal Experimentation at Yamaguchi University School of Medicine and carried out according to the Guidelines for Animal Experimentation at Yamaguchi University School of Medicine and The Law (number 105) and Notification (number 6) of the Japanese Government.

### 2.1. Animals

Male Wistar rats, 280–360 g, were used. They were maintained on a twelve-hour light (7:00–19:00) and dark schedule. Food and water were freely available.

### 2.2. Experimental Paradigm

#### 2.2.1. Pain-Related Response Study

To quantify the pain-related response evoked by MO, the frequency of flinching was counted at 5 min intervals for 60 min after MO (20%, 50 *μ*L) injection into the left hind paw under brief 2% isoflurane anesthesia (MO group: *n* = 6). Control rats received injection of saline (*n* = 6).

#### 2.2.2. Neuronal Activity and Synaptic Transmission Study

*Local Spinal Cord Deoxyglucose Utilization (LSCGU)*. LSCGU was determined according to the method described by Sokoloff et al. [13]. Rats divided into two groups, control group (*n* = 6), and MO group (*n* = 6) were anesthetized with 1.5–2.0% isoflurane for bilateral catheterization of the femoral arteries and veins. Isoflurane was discontinued and each rat was minimally restrained in a specially designed basket. Arterial blood pressure was continuously monitored. Arterial blood gases, pH, and rectal temperature were measured. Body temperature (rectal temperature) was maintained at 37 ± 0.5°C by a heat lamp. Measurements of LSCGU began 15 min after injection of saline or MO into the left hind paw. Rats were transiently anesthetized with 2% isoflurane for intravenous injection of ^14^C-2-deoxyglucose (^14^C-2-DG) followed by light anesthetizing with pentobarbital (50 mg/kg, i.p.) for sedation during the period of LSCGU determination. Arterial blood samples were taken over 45 min, with frequent sampling during the early period in order not to miss the peak of the arterial concentration curves. Blood samples were centrifuged and plasma was separated, followed by freezing samples at −40°C for later determination of ^14^C-2-DG and plasma glucose concentrations. Immediately after the final arterial blood sample collection, 45 min after MO injection, each rat was killed by an i.v. overdose injection of pentobarbital and decapitated. In order to analyse glucose utilization and ligand binding (see below), the spinal cord was widely removed and then lumber spinal cord (L_3–6_) was frozen in isopentane with freon (−50°C). After serial sectioning (20 mm in thickness), spinal cord tissue sections were exposed to X-ray film (SB-5, Kodak) for 10 days, along with a set of calibrated ^14^C standards (Amersham, Japan). Other consecutive tissue sections were used for alter receptor binding study (see below). Local tissue ^14^C concentrations were determined from calibrated ^14^C standards and from optical density measurements made with a computerized image-processing system (UHG-100S1, Unique Medical, Osaka, Japan). Plasma glucose concentrations were determined by the oxidase method using the Beckman Glucose Analyzer 2. The plasma ^14^C-2-DG concentrations were determined in a liquid scintillation counter (Tricab 4640, Packard, USA). The LSCGU was calculated according to the formula described by Sokoloff et al. [13].

^*3*^ *H-PDBu Binding*. ^3^H-PDBu binding was carried out as described by Worley et al. [14]. Tissue sections were prepared from the same animal and then were incubated for 60 min at 23°C in a solution of 50 mM Tris-HCl (pH 7.7), 100 mM NaCl, 1 mM CaCl_2_, and 2.5 nM ^3^H-PDBu (13.2 Ci/mmol, Amersham, Japan). Nonspecific binding was assessed in the presence of 1 mM PDBu. Following incubation, sections were washed twice at 4°C. Tissue sections were dried under a stream of cold air and exposed against ^3^H-Hyper film (Amersham) for 10 days. Quantitative analysis of ^3^H-PDBu binding was performed in essentially the same manner as described above for sP binding.

*Statistical Analysis*. All data were expressed as mean ± SEM. For comparisons between control and MO groups and between “premeasurement” and “measurement”, two-way analysis of variances (two-way ANOVA) followed by Tukey's multicomparison was performed for physiological variables, LSCGU and ^3^H-PDBu binding. Statistical significance was considered when *P* < 0.05.

## 3. Results

### 3.1. Pain-Related Response

**Figure 1:**
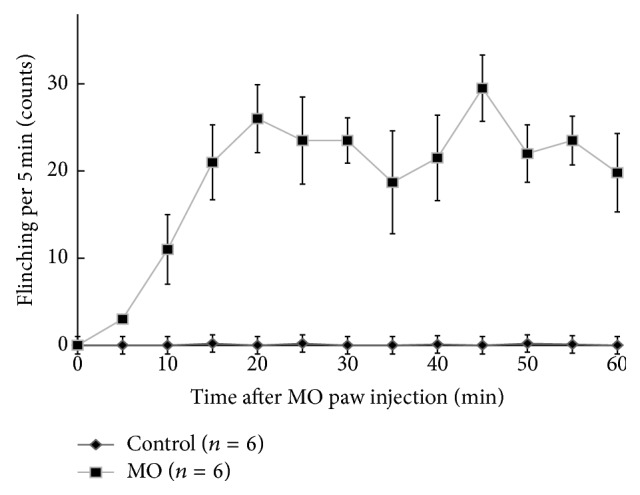
Time course of flinches following mustard-oil paw injection in rats. The flinching behavior increased with time after mustard-oil paw injection. For the following synapse transmission study, rats were perfused and fixed at 60 min after MO injection.

Control animals (control group: saline paw injection for MO injection) showed no indication of flinching during a 60 min period. In the MO group, MO paw injection evoked an increase in flinching. This increase was steadily significant from 15 min (average: 22) to 60 min (average: 21) after MO paw injection (Figure 1).

### 3.2. Changes in LSCGU

Physiological variables during LSCGU determination are shown in Table 1. There was statistical significance between two groups but remained in the normal range for rats.  Table 2 shows that MO paw injection (MO group) increased LSCGU by 18% in Rexed I-II and 14% in Rexed V-VI of the ipsilateral spinal cord compared to the control group.

### 3.3. Changes in Binding for ^3^H-PDBu

**Figure 2:**
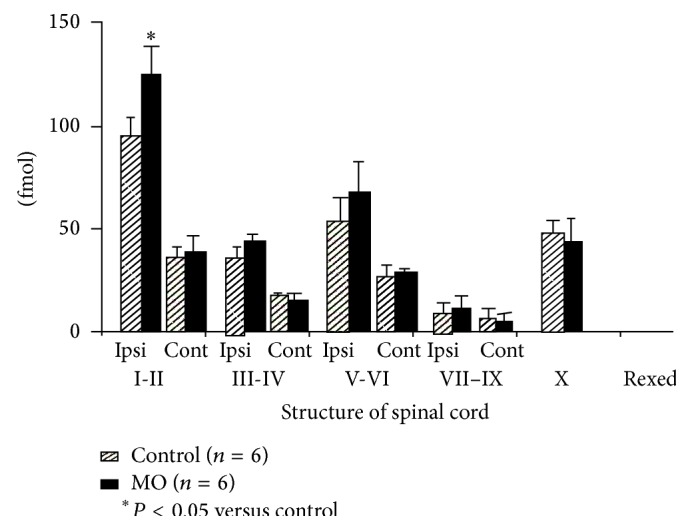
Changes in ^3^H-PDBu binding sites during hyperalgesia in rats. Dashed columns indicate a control group and the black column indicates mustard oil injection (in saline) group. ^*^ *P* < 0.05 versus control group. This binding site is briefly reflected as a translocation to membrane site of protein kinase C activity because of its activator. Mustard-oil injection increased ^3^H-PDBu at Rexed I, with the input site of stimulation from the given peripheral site, as well as sP binding sites.

After MO paw injection, ^3^H-PDBu bindings significantly increased by 22% in Rexed I-II in the ipsilateral side of spinal cord compared to those of control group. In the contralateral side, no significant change compared to that of control group was observed (Figure 2).

## 4. Discussion

This study was clearly conducted to characterize the spatial distribution of neuronal activation related to signaling in the spinal cord in MO-produced inflammatory pain. This is consists of flinching “hyperalgesia” using ^14^C-deoxyglucose (LSCGU) method and *in vitro* ^3^H-PDBu binding sites. The hyperalgesic state was primarily observed with increased LSCGU (neuronal activation) in the Rexed I-II of the spinal cord associated with increased ^3^H-PDBu (PKC: signaling) binding sites.

Recent investigations have revealed that hyperalgesia is developed when the elevated glutamate/sP release between neurons and glial cells (the elevated release of nitric oxide, ILs and other cytokines, and PGE_2_, which trigger microglial activation) increases the efficiency of neurotransmission. Woolf and Salter [2] have suggested that neuronal plasticity in spinal synaptic transmission is the primary mechanism behind persisting spontaneous pain and hyperalgesia following peripheral tissue injury. The results of plentiful research conducted subsequent to this proposal suggest that PKC, MAPKs (ERK1/2, p38-MAPK, and JNK) [3, 4], and BDNF derived from glial cell activation contribute to “neuronal plasticity” [15–17]. A variety of intracellular signaling processes including PKC contribute to progression of chronic pain. Thus, this study revealed further understanding of neuronal pathways of nociceptive information along with cell response in a discrete region of spinal cord in inflammatory pain.

### 4.1. Local Neuronal Activation during Hyperalgesia

Since values obtained by the ^14^C-2-DG method are influenced by neuronal activity, the changes induced by injection of MO in LSCGU may represent synaptic activation in the spinal cord [13]. Increases of LSCGU in Rexed I-II and V-VI indicate that nociception induced by MO is transmitted to the dorsal horn of the spinal cord, particularly to Rexed I-II followed by Rexed V-VI. The present results were similar to metabolic/neuronal changes observed after injection of formalin [18], peripheral nerve stimulation [19], and noxious thermal stimulation [20]. This elevation of neuronal/metabolic activity reflects increased neuronal activity produced by giving noxious peripheral nerve stimulation.

This noxious stimulation activates the C fiber, in which sP and glutamate are known to be major transmitters of pain transmission in the spinal cord [8]. For example, intrathecal injection of substance P (sP) or NMDA evokes activation of dorsal horn neurons associated with hyperalgesia [21]. It is well-known that NMDA receptor inhibitor attenuates these changes in neurotransmitter release in developing hyperalgesia [8], suggesting that these analgesic effects may be beneficially inhibiting presynaptic nerve activity during peripheral tissue inflammation. This concept is similar to that previously investigated by Malmberg and Yaksh [8] who measured spinal cord sP and PGE_2_ release.

### 4.2. Intracellular Signaling and PKC Activation in Spinal Cord

In our previous study, an increase in CSF-glutamate level was observed in association with an increase in sustained pain behavior [22]. This observation indicates that a massive release of spinal glutamate occurred from C fiber terminals at Rexed I-II. On the other hand, it cannot exclude that there is a possibility of an internalization of sP complex into a cytosolic component.

Increased spinal glutamate release due to the activation of C fiber leads to increases in NMDA receptor activation located at Rexed I-II, thereby opening voltage dependent Ca channels and leading to increased turnover of phospholipase C (PLC), followed by increased diacylglycerol (DG), a precursor of PKC [12, 23]. Nakanishi et al. [10] have demonstrated that a PKC inhibitor significantly suppresses pain behavior concomitant with decreased cerebrospinal glutamate release evoked by formalin paw injection. These changes activate the PKC enzyme in the cytosol. Thus, the profound increase in ^3^H-PDBu binding sites indicates that activation of PKC occurred after MO paw injection which was much higher levels of binding than that of saline injection. It may be reasonable that ^3^H-PDBu binding is known to reflect PKC activation/translocation from cytosolic to membrane sites.

In conclusion, the present data confirm that C fiber input activates the superficial layer (Rexed I-II) followed by activation of dorsal horn neurons (Rexed V-VI) which leads to a facilitated pain state after peripheral injury. Moreover, this activation of C fibers may lead to massive release of sP, resulting in a down-regulation of the NK-1 receptor. Activation/translocation of PKC by substance P may be related to functional changes in the central nervous system, constituting “neuronal plasticity” and may result in increased neuronal excitability and a central hyperactive state [2].

## 5. Conclusion

The peripheral injection of mustard oil produced sustained pain-related flinching behavior. This hyperalgesic state was mediated by an increase in LSCGU in Rexed I-II, associated with an increase in ^3^H-PDBu binding. Thus, these data confirmed that C fiber input activates the Rexed I-II followed by activation of dorsal horn neurons (Rexed V-VI) which leads to a facilitated pain state after peripheral tissue injury. In addition, functional changes in the central nervous system represent “neuronal plasticity” and may result in increased neuronal excitability and a central sensitization.

## Figures and Tables

**Table 1:** Physiologic variables.

Stage	Variables	Control (*n* = 6)	MO (*n* = 6)
Premeasurement	MAP (mmHg)	141 ± 5	130 ± 4
	PaO_2_ (mmHg)	146 ± 23	185 ± 40
	PaCO_2_ (mmHg)	48 ± 1	48 ± 1
	pHa	7.36 ± 0.01	7.35 ± 0.01
	Blood sugar (mg/dL)	150 ± 8	152 ± 6
	Rectal temperature (°C)	36.7 ± 0.2	37.2 ± 0.2

During measurement	MAP (mmHg)	130 ± 10	132 ± 4
	PaO_2_ (mmHg)	189 ± 35	266 ± 30
	PaCO_2_ (mmHg)	48 ± 1	51 ± 1^∗#^
	pHa	7.34 ± 0.01	7.30 ± 0.01^*^
	Blood sugar (mg/dL)	140 ± 8	186 ± 12^∗#^
	Rectal temperature (°C)	37.1 ± 0.1	36.8 ± 0.2

Mean ± SEM.

∗Significant versus control (*P* < 0.05).

^#^Significant versus premeasurement (*P* < 0.05).

**Table 2:** Changes in local spinal cord glucose utilization (*μ*g/100 g/min).

Structures	Control (*n* = 6)		MO (*n* = 6)	
Rexed	Contralateral	Ipsilateral	Contralateral	Ipsilateral
I-II	31 ± 1	31 ± 1	31 ± 1	37 ± 1^∗#^
III-IV	34 ± 0	34 ± 0	34 ± 1	35 ± 0
V-VI	37 ± 1	37 ± 0	40 ± 1	42 ± 1^*^
VII–IX	37 ± 1	36 ± 1	38 ± 2	40 ± 1
X	37 ± 1		39 ± 1	

Mean ± SEM.

∗Significant versus control (*P* < 0.05).

^#^Significant versus contralateral side (*P* < 0.05).
